# Downregulation of Smurf2, a tumor-suppressive ubiquitin ligase, in triple-negative breast cancers: Involvement of the RB-microRNA axis

**DOI:** 10.1186/1471-2407-14-57

**Published:** 2014-02-03

**Authors:** Xianpeng Liu, Xin Gu, Limin Sun, Ashley B Flowers, Alfred W Rademaker, Yiran Zhou, Hiroaki Kiyokawa

**Affiliations:** 1 Department of Molecular Pharmacology & Biological Chemistry, Northwestern University, Chicago, IL 60611, USA; 2 Department of Pathology, Louisiana State University Health Science Center, Shreveport, LA, USA; 3 Preventive Medicine, Northwestern University, Chicago, IL 60611, USA; 4 Robert H Lurie Comprehensive Cancer Center, Northwestern University, Chicago, IL 60611, USA

**Keywords:** Triple-negative breast cancer, Ubiquitination, MicroRNA, Retinoblastoma, Tumor suppressor

## Abstract

**Background:**

The HECT family ubiquitin ligase Smurf2 regulates cell polarity, migration, division, differentiation and death, by targeting diverse substrates that are critical for receptor signaling, cytoskeleton, chromatin remodeling and transcription. Recent studies suggest that Smurf2 functions as a tumor suppressor in mice. However, no inactivating mutation of *SMURF2* has been reported in human, and information about Smurf2 expression in human cancer remains limited or complicated. Here we demonstrate that Smurf2 expression is downregulated in human breast cancer tissues, especially of the triple-negative subtype, and address the mechanism of Smurf2 downregulation in triple-negative breast cancer cells.

**Methods:**

Human breast cancer tissues (47 samples expressing estrogen receptor (ER) and 43 samples with triple-negative status) were examined by immunohistochemistry for the expression of Smurf2. Ten widely-studied human breast cancer cell lines were examined for the expression of Smurf2. Furthermore, microRNA-mediated regulation of Smurf2 was investigated in triple-negative cancer cell lines.

**Results:**

Immunohistochemical analysis showed that benign mammary epithelial cells expressed high levels of Smurf2, so did cells in ductal carcinomas in situ. In contrast, invasive ductal carcinomas showed focal or diffuse decrease in Smurf2 expression, which was observed more frequently in triple-negative tumors than in ER-positive tumors. Consistently, human triple-negative breast cancer cell lines such as BT549, MDA-MB-436, DU-4475 and MDA-MB-468 cells showed significantly lower expression of Smurf2 protein, compared to ER + or HER2+ cell lines. Studies using quantitative PCR and specific microRNA inhibitors indicated that increased expression of miR-15a, miR-15b, miR-16 and miR-128 was involved in Smurf2 downregulation in those triple-negative cancer cell lines, which have mutations in the retinoblastoma (*RB*) gene. Forced expression of RB increased levels of Smurf2 protein with concomitant decreases in the expression of the microRNAs.

**Conclusions:**

This study provides evidence of posttranscriptional downregulation of Smurf2 in triple-negative breast cancers, and demonstrates that the loss of RB function is involved in microRNA-mediated interference with Smurf2 translation. The new link from RB inactivation to Smurf2 downregulation is likely to play a role in malignant phenotypes of triple-negative breast cancer cells.

## Background

Triple-negative breast cancers (TNBCs), which lack the expression of estrogen receptor (ER) and progesterone receptor (PR) and the amplification of the *HER2* gene, are a clinically aggressive and molecularly diverse type of breast cancer [1]. TNBCs constitute 10%–20% of all breast cancers and highly prevalent in African-American women [2]. The survival rates of breast cancer patients have shown a tendency of improvement recently, possibly owing to targeted therapies against ER/PR-positive or HER2-positive cancers. Nonetheless, the treatment of patients with TNBC remains to be a major challenge, and TNBC is associated with poorer prognosis than other breast cancer subtypes [3]. A recent study demonstrated that TNBCs can be categorized into at least six subgroups based on the gene expression profiles [1]. Profiling the transcriptomes of cancer tissues and cell lines has significantly advanced our knowledge in the biology of TNBC and potential therapeutic targets; however, it remains obscure how posttranscriptional changes in tumor suppressors or oncoproteins contribute to the development of TNBC.

Smurf2 is a HECT-family ubiquitin ligase (E3), which has been implicated in diverse biological functions including the transforming growth factor-beta (TGF-β) signaling, mitotic regulation, cell polarity, motility and chromatin modifications [4]. According to the literature, Smurf2 appears to play complex roles in tumorigenesis. A previous study using immunohistochemistry showed that esophageal squamous cell carcinomas expressed high levels of Smurf2, which correlated with poor prognosis [5]. Another study on lung adenocarcinomas and head & neck carcinomas showed a positive correlation between Smurf2 protein levels and EGFR protein levels [6]. In contrast, there have been several reports demonstrating decreased expression of Smurf2 in other types of cancer. Protein levels of Smurf2 were found to be downregulated in human lymphoma and breast cancer tissues relative to non-cancer tissues [7]. In a study on prostate cancers, Smurf2 mRNA levels were lower in advanced tumors compared to less advanced organ-confined tumors, suggesting association of Smurf2 downregulation with tumor progression [8]. Importantly, two recent studies using *Smurf2*-null mice have shown that Smurf2-deficiency increases susceptibility to spontaneous tumorigenesis in various tissues including the liver, lung, pituitary and mammary gland [7,9]. The activity of Smurf2 to ubiquitinate and degrade RNF20, a RING-family E3 that controls histone H2B ubiquitination and genome stability, has been implicated for the tumor suppressive role of Smurf2 [7].

In this study we demonstrate that human TNBC tissues express significantly lower levels of Smurf2 protein relative to normal mammary tissues, ductal carcinomas in situ (DCIS) and ER+/PR + breast cancer tissues. We also have revealed that microRNAs such as miR-15a, miR-15b, miR-16 and miR-128, whose expression is increased by inactivating mutations of the retinoblastoma (*RB*) gene, downregulate translation of Smurf2 protein in TNBC cells. These results suggest that Smurf2 downregulation is an event associated with RB loss and microRNA deregulation during the progression of TNBC, and likely involved in the aggressive phenotypes.

## Methods

### Patients

Surgical specimens were obtained from breast cancer patients (47 ER+/PR + and 43 TN) who had mastectomy or lumpectomy at Louisiana State University Health Sciences Center, Shreveport, LA, during the period between 2002 and 2010. This study was reviewed and approved in advance by the Institutional Review Boards of the Louisiana State University Health Sciences Center and the Feinberg School of Medicine, Northwestern University. All necessary consent was obtained from every patient involved in the study, including consent for participation in the study and publication of data. The patients’ ages ranged from 27 to 96 years, and their mean age was 54.2 years. Tumor stages were classified according to the seventh edition of the TNM (tumor-node-metastasis) classification of breast carcinomas published by American Joint Committee on Cancer. The clinicopathological parameters of the patient cohorts are shown in Table 1 and Additional file 1: Table S1.

**Table 1:** Clinicopathologic characteristics of cohorts of breast cancers

	**ER/PR +**	**TN**
**No. of cases**	**47**	**43**
Tumor morphology	37	39
Ductal	6	1
Lobular	3	
Mucinous	1	
Tubular		2
Medullary		1
Metaplastic		
Her-2/Neu	9	0
Overexpressed	38	43
Not overexpressed		
Elston grade	7	2
Grade 1	35	14
Grade 2	5	27
Grade 3		
TMN stage at first diagnosis	15	10
I	18	20
II	14	12
III	0	1
IV		
Lymph node status ar diagnosis	25	17
Positive	18	26
Negative	4	0
Not assessed		
Metastasis	0	1

### Immunohistochemistry for Smurf2

**Figure 1:**
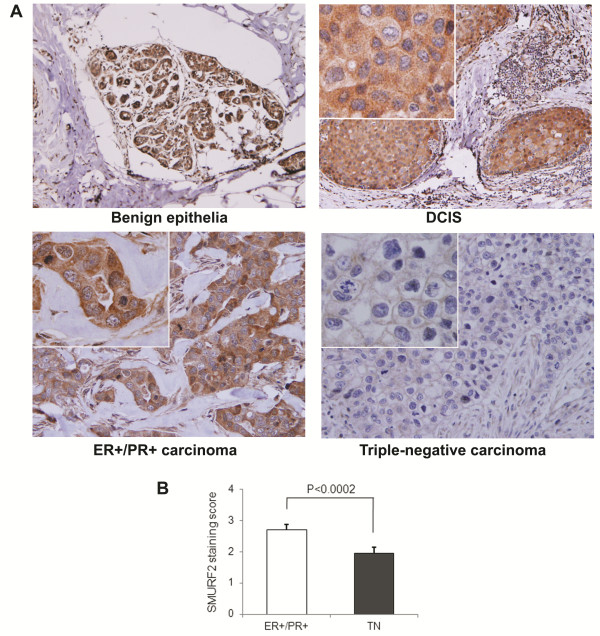
**Smurf2 expression is decreased in triple-negative breast cancer tissues. (A)** Immunohistochemistry for Smurf2 was conducted using human mammary tissues including benign mammary epithelia, ductal carcinomas in situ (DCIS), ER+/PR + and triple-negative (TN) invasive carcinomas. Upper left panel shows x10 magnification, and the other panels show x20 pictures with x40 magnified views. **(B)** Percent of Smurf2-positive cells in ER+/PR + (n = 47) and TN (n = 43) breast cancer specimens. To quantify focal loss of Smurf2 expression in carcinoma regions, immunohistochemical labeling of Smurf2 was scored according to the following five categories: 0 (Smurf2^+^ cells in carcinoma tissues: 0-5%), 1+ (6%-25%), 2+ (25%-50%), 3+ (50%-75%), and 4+ (>75%). Data are shown as mean + SEM with the p value for statistical significance.

Immunohistochemical staining of paraffin-embedded human tissues was performed by the standard avidin-biotin peroxidase complex method. Paraffin sections were labeled and dried in 60°C oven for at least 4 hour, cooled, deparaffinized, and incubated in antigen retrieval solution (#H-3300; Vector Laboratories, Burlingame, CA). For antigen retrieval, slides were heated and cooled in antigen retrieval solution for 25 and 20 minutes, respectively. Slides were then rinsed 4–5 times in distilled water; once in 0.3% peroxide in 50% methanol for 30 minutes, and 3 times for 5 minutes in wash buffer. Subsequently, slides were processed using the BioGenex i6000 Automated Staining System. Blocking was conducted by soaking slides in 10% goat serum in phosphate-buffered saline (PBS), for 15 minutes, in 5% casein block in PBS for 10 minutes, and in 10% goat serum in PBS for 1 minute. Slides were then incubated with the primary antibody for Smurf2 (Y-21: sc-130878, Santa Cruze Biotechnology, CA) at a dilution of 1:100 in Dako antibody diluent (Dako, Glostrup, Denmark) for 1 hour, followed by 5 times rinse with wash buffer. Samples were then incubated with the secondary (MultiLink-BioGenex Super Sensitive Link-Label IHC Detection System #QP900-9 L; BioGenex, Fremont, CA) for 20 minutes, rinsed 3 times in wash buffer, and labeled with a horseradish peroxidase solution (BioGenex Super Sensitive Link-Label IHC Detection System #QP900-9 L) for 15 minutes. Following triple washes, 3,3-Diaminobenzidine (#K3466; DakoCytomation Liquid DAB Substrate Chromogen System) was applied to samples for 5 minutes. Samples were then rinsed 3 times, stained with hematoxylin (#S3301; DakoCytomation Automation Hematoxylin) for 2 minutes, and rinsed 3 times again in wash buffer. Slides were then rinsed with distilled water for 4 minutes, and dehydrated sequentially with ethanol and xylene. A negative control to each section was prepared by using normal rabbit serum instead of the primary antibody. While benign mammary epithelia and ductal carcinomas in situ (DCIS) displayed uniform strong staining for Smurf2, invasive carcinomas often exhibited focal patterns of Smurf2 staining (see Figure 1). To comparatively examine decreased expression of Smurf2 in invasive carcinomas, percentages of Smurf2-positive cells in carcinoma regions were scored as follows: 0 (Smurf2^+^ cells in carcinoma tissues: 0-5%), 1+ (6%-25%), 2+ (25%-50%), 3+ (50%-75%), and 4+ (>75%).

### Cell culture and reagents

Human non-transformed mammary epithelial MCF-10A cells, and 9 human breast cancer cell lines, MCF-9, T47D, MDA-MB-231, BT549, MDA-MB-436, DU4475, MDA-MB-468, BT474 and SK-BR-3, were obtained from American Tissue Culture Collection (ATCC), and cultured under standard conditions recommended by ATCC. Fetal bovine sera and calf sera were obtained from HyClone/Thermo Fisher Scientific (Logan, UT), and media, antibiotics and other chemicals were purchased from Corning Cellgro (Manassas, VA) and GiBCO/Invitrogen (Carlsbad, CA). Cycloheximide was purchased from Sigma-Aldrich (St. Louis, MO).

### Immunoblotting

Immunoblotting was performed as previously described [10]. Anti-Smurf2 antibody was obtained from Upstate Biotechnology (07–249, Lake Placid, NY), and anti-RB and anti-α-Tubulin antibodies were from BD Pharmingen (554136, BD Biosciences, San Jose, CA) and Sigma-Aldrich (T6199), respectively. Target proteins were visualized by enhanced chemiluminescence (Thermo Scientific, Rockford, lL). The band intensities were quantified by densitometry using the Photoshop and Image J software and normalized to those of their respective control bands.

### Real time PCR

Total RNA samples were collected using the Trizol reagent (Invitrogen, Carlsbad, CA). Levels of Smurf2 mRNA were quantified in comparison with those of GAPDH mRNAs, using the Power SYBR**®** Green PCR Master Mix (Applied Biosystems**,** Carlsbad, CA**)** and the Applied Biosystems 7900. Levels of miR-15a, miR-15b, miR-16 and miR-128 were measured by quantitative RT-PCR, using miScript PCR system including pre-designed miRNA-specific primers (Qiagen, Valencia, CA) and the Applied Biosystems 7900. RNU6-2 was used as the reference endogenous control, and 2^-ΔΔCt^ method [11] was used to analyze the relative miRNA expression.

### Transfection with plasmids and miRNA inhibitors

Cells were transfected with Ambion® Anti-miR™ miRNA Inhibitors specifically against miR-15a, miR-15b, miR-16 and miR-128 (Ambion/Invitrogen, Carlsbad, CA), using the Lipofectamine® RNAiMAX transfection reagent (Invitrogen, Carlsbad, CA) according to the manufacturer’s protocol. The expression vector for green fluorescence protein (GFP) fused with full length retinoblastoma protein (RB) and pEGFP-C3 for GFP expression were obtained from Addgene (Cambridge, MA). Plasmid transfection was conducted with the Lipofectamine® 2000 reagent from Invitrogen, according to the manufacturer’s protocol.

### Statistical analysis

Immunohistochemical labeling of Smurf2 in carcinoma tissues was scored as described above and statistically analyzed using Fisher’s exact test and the Wilcoxon rank sum test. Other quantified data from immunoblotting and real time PCR were analyzed using Student’s *t* test. P < 0.05 was considered significant.

## Results

### Smurf2 downregulation in TNBC

**Figure 2:**
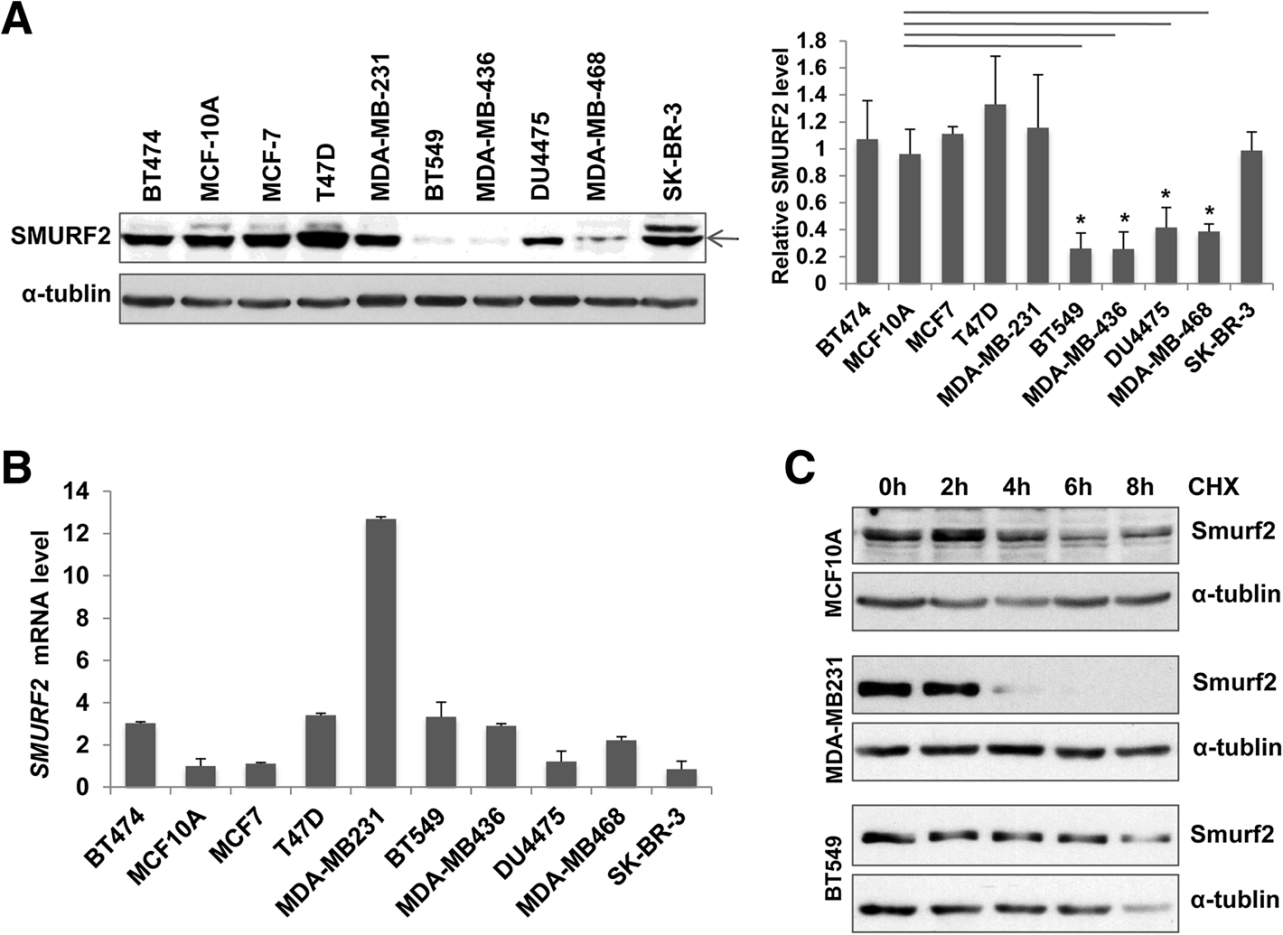
**Smurf2 protein is downregulated in triple-negative breast cancer (TNBC) cell lines without concomitant decreases in Smurf2 mRNA. (A)** Immunoblotting for Smurf2 in the following cell lines: MCF-10A, untransformed human mammary epithelial cells; MCF-7 and T47D, mammary carcinomas with expression of the estrogen and progesterone receptors (ER+/PR+); MDA-MB-231, BT549, MDA-MB-436, DU4475 and MDA-MB-468, TNBCs; BT474 and SK-BR-3, mammary carcinomas with *HER2* amplification. The bar graph indicates relative levels for Smurf2 protein in the cancer cell lines to that in MCF-10A cells. The density of each Smurf2 signal on immunoblots was normalized by that of α-tubulin. Data are shown as mean + SEM from at least three experiments, and the asterisks indicate statistically significant (p < 0.05) differences from the level in MCF-10A cells. **(B)** Real time PCR analysis for Smurf2 mRNA. Data are normalized by signals for GAPDH mRNA, and presented as relative expression levels to that in MCF-10A cells. **(C)** Degradation of Smurf2 protein assessed by treatment with cycloheximide (CHX). MCA-10A, MDA-MB-231 and BT549 cells were treated with 100 μg/ml CHX for the indicated hours, and lysates were analyzed by immunoblotting for Smurf2 and α-tubulin.

To determine whether the expression of Smurf2 protein was altered in breast cancer tissues, surgical specimens from 90 breast cancer patients (47 with ER+/PR + cancers and 43 with TNBCs, see Table 1 and Additional file 1: Table S1) were analyzed by immunohistochemistry for Smurf2. Regions of benign mammary epithelia and DCIS showed robust Smurf2 staining both in the cytoplasm and nucleus (Figure 1A, upper panels). In samples with invasive carcinomas, Smurf2 staining was found decreased focally or sometimes diffusely, and the downregulation of Smuf2 was significantly more obvious in TNBCs compared to ER+/PR + cancers (Figure 1A lower panels, Figure 1B). The median of the Smurf2 staining scores in TNBCs was 2 (25%-50% of tumor cells were Smurf2-positive), while that in ER+/PR + cancers was 3 (50%-75% Smurf2-positive). Higher tumor grades and Ki67 scores were observed in the TN group, compared with the ER+/PR + group. Lower Smurf2 staining scores were associated with higher tumor grades (p = 0.0004) and higher Ki67 scores (p = 0.011), but not with stages or p53 staining scores (Additional file 1: Table S1). We then examined human breast cancer cell lines and non-transformed mammary epithelial MCF-10A cells by immunoblotting for Smurf2 (Figure 2A). Levels of Smurf2 protein in ER+/PR + cancer cells (MCF-7 and T47D) and those in HER2+/ER+/PR + BT474 cells and HER2+/ER-/PR- SK-BR-3 cells were comparable with Smurf2 levels in MCF-10A cells. In sharp contrast, Smurf2 protein levels in 4 of 5 TNBC cell lines, BT549, MDA-MB-436, DU-4475 and MDA-MB-468 cells, were significantly lower than those in MCF-10A and the ER+/PR + cell lines. Only MDA-MB-231 cells showed high levels of Smurf2 expression. To determine whether Smurf2 downregulation in the TNBC cell lines resulted from transcriptional repression, Smurf2 mRNA levels were measured by real time PCR (Figure 2B). In the four cell lines that exhibited lower levels of Smurf2 protein, no decreases in the mRNA levels were observed, relative to that in MCF-10A cells, suggesting that Smurf2 is downregulated at the posttranscriptional level in those TNBC cell lines. In contrast, MDA-MB-231 cells exhibited remarkably higher Smurf2 mRNA levels, indicating that Smurf2 is transcriptionally upregulated only in this particular cell line. To further examine whether protein degradation plays a dominant role in determining the steady-state level of Smurf2 protein, we examined the stability of Smurf2 in MCF-10A, MDA-MB-231, and BT549 cells, using the translation inhibitor cycloheximide (Figure 2C). According to the decay of Smurf2 levels in the presence of cycloheximide, the half-life of Smurf2 in MCF-10A cells was determined to be about 8 hours. Interestingly, the half-life of Smurf2 in MDA-MB-231 cells was less than 3 hours, suggesting that Smurf2 protein is rather more unstable in this cell line that overexpresses its mRNA. On the other hand, Smurf2 protein was more stable in BT549 cells, displaying a half-life of more than 12 hours. Taken together, these data indicated that the expression of Smurf2 protein is downregulated frequently in human TNBC tissues, and similar downregulation was observed in four of the five TNBC cell lines examined here. MDA-MB-231 cells exceptionally showed transcriptional upregulation of Smurf2, which appeared to be counteracted by enhanced degradation of the protein.

### miR-15/16 and miR-128 mediate Smurf2 downregulation

**Figure 3:**
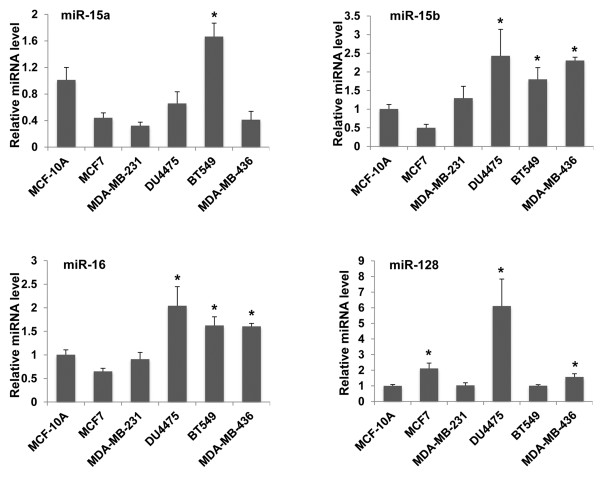
**Expression levels of miR-15a, miR-15b, miR-16 and miR-128 in breast cancer cell lines.** Levels of the indicated microRNAs were determined by real time PCR in the following cell lines: MCF-10A, untransformed human mammary epithelial cells; MCF-7, mammary carcinomas with expression of the estrogen receptor (ER+); MDA-MB-231, BT549, MDA-MB-436, and DU4475, triple-negative mammary carcinomas. Data are normalized by signals for RNU6-2 as controls, and presented as relative expression levels to that in MCF-10A cells as 1.0. The bars indicate S.E.M. from 3–5 experiments, and the asterisk indicates statistically higher (p < 0.05) levels of each miRNA in the particular cell line than in MCF-10A cells.

**Figure 4:**
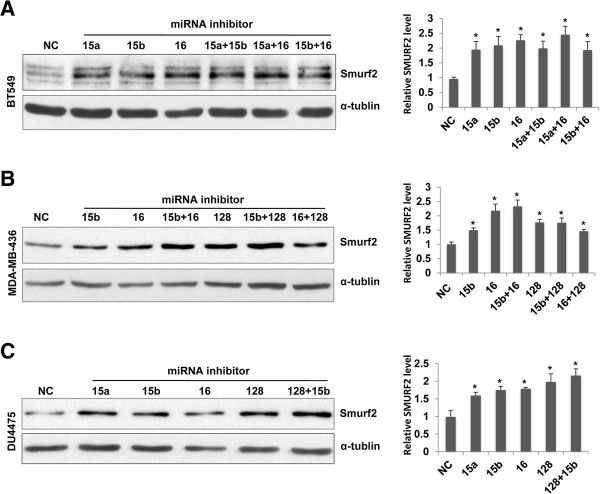
**MicroRNAs such as miR-15, miR-16 and miR-128 are involved in downregulation of Smurf2 protein in triple-negative breast cancer.** Human triple-negative breast cancer cell lines, BT549, MDA-MB-436 and DU4475 cells, were transfected with microRNA inhibitors against miR-15a, miR-15b, miR-16 and miR-128, or nonspecific ssRNA as negative control (NC), and cellular levels of Smurf2 protein were determined at 24 h **(A, B)** or 48 h **(C)** post-transfection by immunoblotting. The density of each Smurf2 signal on immunoblots was normalized by that of α-tubulin, and presented as relative expression levels to that in negative control (NC) as 1.0. Quantified data on bar graphs show means + SEM from three experiments, and the asterisks indicate statistically significant (p < 0.05) differences from negative control (NC).

Deregulation of microRNAs (miRNAs) has been implicated to the biology of breast cancer such as estrogen signaling, migration and metastasis [12,13]. We hypothesized that some miRNAs were involved in the post-transcriptional downregulation of Smurf2 in TNBC, and used multiple online databases such as TargetScan and PicTar to identify miRNAs that potentially bind to Smurf2 mRNAs. The analysis led us to candidates such as miR-128 (binding to Smurf2 3′UTR, 5′-CACUGUGA-3′) and the miR-15 family miRNAs including miR-15a, miR-15b and miR-16 (binding to Smurf2 3′UTR, 5′-GCUGCUA-3′). The miR-15 family and miR-128 have been implicated for the regulatory network in breast cancer initiating cells [14,15]. Thus, we measured the expression of miR-15a, miR-15b, miR-16 and miR-128b in the breast cancer cell lines (Figure 3). DU4475 cells showed increased expression of miR-15b, miR-16 and miR-128, relative to their expression in MCF-10A cells. BT549 cells exhibited increased expression of miR-15a, miR-15b and miR-16. MDA-MB-436 cells had increased expression of miR-15b, miR-16, and miR-128. Thus, these TNBC cell lines that exhibited Smurf2 downregulation had a tendency to express higher levels of these miRNAs. In contrast, MDA-MB-231 cells, which had high levels of Smurf2 mRNA and protein, showed no major change in the expression of these miRNAs, except for a decrease in miR-15a. Also in MCF-7 cells, the levels of miR-15a, miR-15b and miR16 were low, whereas the expression of miR-128 was modestly higher. To further delineate the role of the miRNAs in Smurf2 downregulation observed in BT549, MDA-MB-436 and DU4475 cells, cells were transfected with miRNA inhibitors (antagomirs) against miR-15a, miR-15b, miR-16 or miR-128 (Figure 4). Treatment with these antagomirs resulted in substantial increases in Smurf2 protein levels in the TNBC cell lines, suggesting the involvement of these miRNAs in downregulating Smurf2 in TNBC.

### Linkage of RB mutations to miRNA deregulation and Smurf2 downregulation

**Figure 5:**
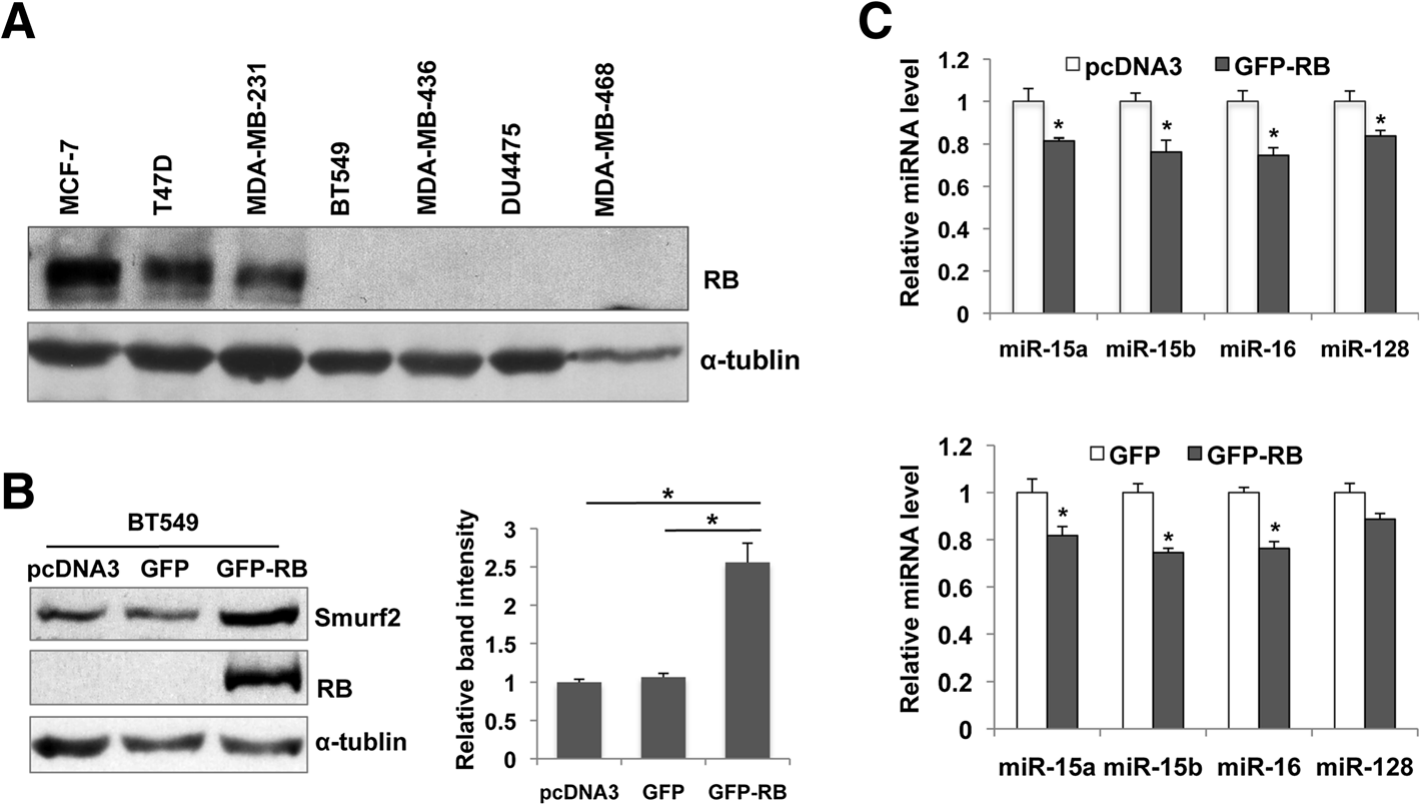
**The loss of the retinoblastoma tumor suppressor (*RB*) expression plays a role in Smurf2 downregulation in triple-negative breast cancer (TNBC) cells, via upregulation of miR-15, miR-16 and miR-128. (A)** Immunoblotting for RB protein in the indicated TNBC cell lines. **(B)** Increased expression of Smurf2 by forced expression of RB in *RB-*null BT549 cells. Cells were transfected with an expression vector for a green fluorescence protein (GFP)-RB fusion protein, a vector for GFP, or an empty vector (pcDNA3.1). Levels of Smurf2 and RB were determined at 42 h post-transfection by immunoblotting. The bar graph indicates relative levels for Smurf2 protein normalized by those for α-tubulin, shown as means + SEM from three experiments. The asterisk indicates statistical significance (p < 0.05). **(C)** Decreased expression of miR-15a, miR-15b, miR-16 and miR-128 in BT549 cells transfected with the GFP-RB vector. Levels of the indicated microRNAs were determined by real time PCR. Data are normalized by signals for RNU6-2 as controls, and presented as relative expression levels to that in cells transfected with an empty vector as 1.0. The bars indicate S.E.M. from three experiments, and the asterisk indicates statistical significance (p < 0.05).

A recent study demonstrated that miR-15 and miR-16 are direct targets of the E2F transcription factors [16]. A number of TNBCs have inactivating mutations of the retinoblastoma tumor suppressor gene (RB) [17], which lead to hyperactivation of E2F [18]. Therefore, we hypothesized that RB inactivation could result in elevated expression of the miR-15 family and possibly miR-128, which contributed to the downregulation of Smurf2. Immunoblotting for RB demonstrated that all four TNBC cell lines that exhibited Smurf2 downregulation had no detectable expression of RB (Figure 5A). In contrast, MDA-MB-231 cells, which expressed high levels of Smurf2, showed robust RB expression comparable to that in MCF-7 and T47D cells. This RB expression patterns are consistent with the genotypes of the *RB* gene in these cell lines as summarized in [1]. To further examine the role of RB in the regulation of Smurf2, we transfected BT549 cells with an expression vector for the full-length RB protein fused with green fluorescence protein (GFP) (Figure 5B). Forced expression of GFP-RB resulted in a significant increase in cellular levels of Smurf2 protein, accompanied by substantial decreases in the expression of miR-15a, miR-15b, miR-16 and miR-128b (Figure 5C). These results indicate that forced expression of RB in TNBC cells with RB mutations could restore levels of Smurf2 protein expression, suggesting the significance of the RB-miRNA pathway in the control of Smurf2 in TNBC.

## Discussion

Here we present evidence that the expression of Smurf2 protein is downregulated preferentially in TNBC. The cancer-associated downregulation is consistent with the recent studies that suggested the tumor suppressive function of this E3 enzyme [7,9]. Low expression of Smurf2 protein was also observed in several TNBC cell lines, which had *RB* mutations and high expression of miR-15a, miR-15b, miR-16 and miR-128. Antagomirs against these miRNAs substantially increased Smurf2 levels in the TNBC cell lines. Moreover, forced expression of RB in the TNBC cells increased cellular levels of Smurf2, with concomitant decreases in the expression of those miRNAs. Therefore, RB inactivation accounts at least partly for Smurf2 downregulation in the TNBC cells, via deregulated expression of the miR-15 family and miR-128.

Recent progress in the field has indicated that numerous miRNAs play major roles in breast cancer biology, from tumor initiation to metastasis [13]. Our finding that miR-15/16 and miR-128 are involved in Smurf2 downregulation in TNBC provides a new pathway to the miRNA-mediated biological processes in breast cancer. It was previously demonstrated that miR-15 and miR-16 are direct transcriptional targets of E2F-1, and these miRNAs in turn restrict E2F activities [16,19]. Whereas deletion of miR-15a and miR-16 was reported in some non-small cell lung cancers [19], miRNA expression profiling in human breast cancer subtypes showed that basal-like TNBCs expressed higher levels of miR-15b than other subtypes [20]. This is consistent with our data on the TNBC cell lines. High expression of miR-128 has been associated with poor prognosis of ER + breast cancer [21]. miR-128 is known to target Bmi1, the polycomb transcription factor required for stemness [15,22], and miR-128 expression may be increased during the transition from the cancer-initiating cell state to the expansive state of breast cancer. Interestingly, oncogenic p53(R175H) mutant induces the transcription of miR-128, which then promotes chemoresistance of non-small cell lung cancer [23], presenting another example of high miR-128 expression associated with malignant phenotypes.

Smurf2 is known to be a negative regulator of the TGF-β signaling, as the Smurf2-Smad7 complex ubiquitinates the type I TGF-β receptor and the Smad-associated co-repressor SnoN, targeting them to proteasomal degradation [24,25]. It is now recognized that the TGF-β signaling plays dual roles in the development of breast cancer [26,27]. At the phase of tumor initiation TGF-β functions as a tumor suppressor, inhibiting cell cycle progression during transformation. In contrast, at the late phase of tumor progression TGF-β promotes invasion and metastasis of breast cancer. The cellular context of cancer, in concert with tumor microenvironment, seems to determine the responses to TGF-β signaling, while the exact molecular mechanisms behind the functional transition remain to be elucidated. The downregulation of Smurf2 protein observed in TNBC may contribute to enhanced TGF-β signaling leading to tumor invasion, epithelial-mesenchymal transition and metastasis. Besides the TGF-β signaling components, Smurf2 interacts with a diverse array of proteins, some of which affect tumorigenesis. For example, Smurf2 interacts with MDM2/HDM2 and enhances its ability to ubiquitinate and degrade the tumor suppressor p53 [28], implying that Smurf2 could promote tumorigenesis in some context. On the other hand, Smurf2 targets the helix-loop-helix transcription regulator Id1 (inhibitor of differentiation or DNA binding) for proteasomal degradation [29]. Id1 plays oncogenic roles in inhibiting cellular senescence and maintaining stemness and also in tumor re-initiation during breast cancer metastasis to the lung [30,31]. Many of basal-like TNBCs have loss-of-function mutations in the *RB* gene [17], which may enhance the Id1 functions by downregulating Smurf2. It should be noted that MDA-MB-231 cells, which are TNBC with intact *RB* function, express markedly high levels of Smurf2 mRNA and modestly increased levels of the protein with rapid turn-over. It has been controversial whether Smurf2 promotes or inhibits migration and invasion of TNBC [32,33]. Our study suggests that among widely-used TNBC cell lines, MDA-MB-231 cells are unique with regard to Smurf2 regulation and perhaps its role in tumor progression. The exact impact of Smurf2 downregulation on the development of *RB-*deficient TNBC awaits further investigations.

Increased susceptibility of *Smurf2*-null mice to spontaneous tumorigenesis has provided key evidence for the tumor suppressive actions of Smurf2 [7,9]. Lymphomas and hepatocellular carcinomas are tumor types most commonly observed in two independent strains of *Smurf2*-null mice, while a few percent of *Smurf2*-null mice develop mammary carcinomas [7]. *Smurf2*-null mouse embryonic fibroblasts (MEFs) exhibit impaired senescence responses, and undergo spontaneous transformation more frequently in culture. Genomic instability has been observed in *Smurf2*-null MEFs, together with chromatin compaction associated with increased ubiquitination of histone H2B. These changes seem to be linked with stabilization of the histone ubiquitin ligase RNF20, as Smurf2 usually promotes degradation of RNF20 [7]. Smurf2-deficiency may also result in impaired mitotic regulation and subsequent genomic instability, as demonstrated in several human cancer cell lines with siRNA-mediated silencing of Smurf2 [10]. Taken together, downregulation of Smurf2 in TNBCs with RB mutations could contribute to the malignant phenotypes at multiple levels. Our ongoing study for undefined tumor-suppressive targets of Smurf2 is expected to provide not only novel insight into the biology of TNBC but also candidates for therapeutic targets against this aggressive cancer.

## Conclusions

The present study shows that the HECT-family ubiquitin ligase Smurf2 is downregulated at the posttranscriptional level in many TNBC cells. miRNAs such as miR-15/16 and miR-128, whose upregulation is linked to the inactivation of RB, play important roles in the downregulation of Smurf2. The involvement of Smurf2 in cancer development has been controversial. The new link from RB inactivation to Smurf2 downregulation provides novel insight into the biology of TNBC and potential therapeutic strategies.

## Abbreviations

ER: Estrogen receptor; Id1: Inhibitor of differentiation or DNA binding; miRNA: microRNA; PR: Progesterone receptor; TGF-β: Transforming growth factor-β; TN: Triple-negative; TNBC: Triple-negative breast cancer.

## Competing interests

The authors declare that they have no competing interests.

## Authors’ contributions

XL participated in the design of the study and carried out the microRNA database analysis and cell and molecular biological experiments. XG and ABF conducted immunohistochemistry using human breast cancer tissues and analyzed clinicopathologic data. LS carried out quantitative PCR and data analysis. AWR conducted all statistical analyses. YZ participated in the design of the study and conducted cell biological experiments. HK conceived of the study and took a lead in its design and coordination. All seven authors participated in drafting and revising the manuscript. All authors read and approved the final manuscript.

## Pre-publication history

The pre-publication history for this paper can be accessed here:

http://www.biomedcentral.com/1471-2407/14/57/prepub

## Supplementary Material

Additional file 1: Table S1 Triple-Negative (TN) group. Click here for file
