# Supplementary material for: Downregulation of Smurf2, a tumor-suppressive ubiquitin ligase, in triple-negative breast cancers: Involvement of the RB-microRNA axis
Source: BMC Cancer. 2014 Feb 3;14:57. doi: 10.1186/1471-2407-14-57 (PMC3918234; doi:10.1186/1471-2407-14-57)
Supplement: Additional file 1: Table S1 — Triple-Negative (TN) group. [file 1471-2407-14-57-S1.pdf]

Triple-Negative (TN) group

Case No	ER/PR/Her	Her 2	Ki-67	p53	T, N, M	Stage	Dx	Hist Grade	Score of % cells s	Intensity
1	negative	0.4	80%	87%	pT1b N0	I	IDC	G3	1	2+
2	negative	1	31%	0	T1C, N0	I	IDC	G3	3	2+
3	negative	1.6	79%	0	T1C, N0	I	IDC	G3	0	0+
4	Negative	0	60%	0%	T1, N0	I	IDC	G3	1	3+
5	Negative	0	65	82%	T1b, N0	I	IDC	G2	3	2+
6	Negative	1.8	82%	100%	T1, N0	I	IDC	G1	0	0+
7	Neg	0.5	6%	0%	T1, N0	I	IDC	G2	4	2+
8	Neg	0.5	77%	0%	T1, N0	I	IDC	G3	2	2+
9	Neg	1.6	72%	94%	T1C, Nmic	IB	IDC	G2	2	2+
10	Neg	Neg	19%	42%	T1min, N0	I	IDC	G1	3	3+
11	Neg	0.4	72%	0%	pT1, N2	IIA	IDC	G3	2	2+
12	negative	0.9	75%	95%	pT2, N0	IIA	IDC	G3	2	2+
13	negative	0	0	0	pT2, N0	II	IDC	G2	3	3+
14	negative	IHC 0.9, Po	88%	0	pT2, N0	IIA	IDC	G2	4	2+
15	negative	1.2	36%	46%	T3 N0	IIB	IDC	G3	2	2+
16	negative	1.2	19%	16%	T2 N1	IIB	IDC	G2	4	2+
17	negative	1.8	33%	0	T2 N0	IIA	IDC	G3	1	2+
18	negative	0.5	0	0	T2, N0	IIA	Medulalry C	G3	3	2+
19	Neg	1.3	48%	88%	T2, N0	IIA	IDC	G2	2	3+
20	Negative	0	37%	68%	T2, N0	IIA	IDC	G2	3	3+
21	Negative	0	41%	5%	T2, N1	IIB	IDC	G3	2	3+
22	Negative	1	39%	72%	T2, N0	IIA	IDC	G3	1	2+
23	Negative	1.1	94%	85%	T2, N1	IIB	IDC	G3	1	2+
24	Negative	0.9	68%	0%	T2, N0	IIA	IDC	G3	2	3+
25	Negative	0	20%	19%	T2, N0	IIA	IDC	G3	3	2+
26	Negative	1.7	76%	76%	T2, N1	IIB	Medulalr C	G3	1	2+
27	Neg	2.3	79%	0%	T2, N0	IIA	IDC	G2	2	3+
28	Neg	1	36%	0%	T2, N0	IIA	IDC	G3	1	3+
29	Neg	0	71%	0%	T2, N0	IIA	IDC	G3	2	2+
30	Neg	1.2	92%	94%	T2, N0	IIA	IDC	G3	0	0+

31	negative	0.9	90%	96%	pT3 N2a	IIIA	IDC	G3	4	2+
32	negative	0.6	89%	0	T3, N1	IIIA	IDC	G3	3	2+
33	negative	0.7	15%	3%	T2, N3	IIIC	IDC	G3	3	1+
34	negative	0	78%	87%	T2, N2a	IIIA	IDC	G2	3	2+
35	negative	IHC 1.7, Po	14%	0	T2, N3a	IIIC	IDC	G2	3	2+
36	Negative	0.6	31%	65%	T2, N2	IIIA	IDC, recurr	G3	2	3+
37	Negative	0	0%	70%	T3, N1	IIIA	Metaplastic	G3	0	0+
38	Negative	0.7	9%	68%	T2, N2a	IIIA	IDC	G2	1	3+
39	Negative	0	80%	87%	T4b, N3	IIIC	IDC	G3	1	2+
40	Negative	1.9	83%	85%	T2, N2a	IIIA	IDC	G2	2	3+
41	Neg	0	46%	0%	T4, N2	IIIB	IDC	G3	1	2+
42	Neg	0.8	90%	76%	T3, N2a	IIIA	IDC	G2	0	0+
43	negative	0.7	0	0	pT2 N0 M1	IV	ILC	G3	1	1+

ER+/PR+ group

Case No	ER/PR/Her	Her 2	Ki-67	p53	T, N, M	Stage	DX	Hist Grade	Score of % cell	Intensity
1	Positive	Neg	2%	3%	T1b, N0	I	IDC	G2	2	3+
2	Positive	Neg	5%	0%	T1, N0	I	Coloid	G1	4	3+
3	Positive	Neg	86%	15%	T1, N0	I	IDC	G3	4	2+
4	Positive	Neg	7%	6%	T1, N0	I	IDC	G2	3	3+
5	Positive	Neg	13%	16%	T1, N0	I	IDC	G2	4	3+
6	Positive, 98	Neg	0%	0%	T1, Nx	I	ILC	G2	3	3+
7	Positive	Neg	11%	21%	T1min, N0	I	IDC	G1	3	3+
8	Positive	Neg	9%	0%	T1c, N0	I	IDC	G2	1	2+
9	Positive	Neg	10%	6%	T1, N0	I	ILC	G1	3	3+
10	Positive	Positive	14%	17%	T1c, N0	I	IDC	G2	3	3+
11	Positive	Neg	15%	5%	T1, Nx	I	IDC	G1	3	3+
12	Positive, 81	1.6	15%	0%	T1, N0	I	IDC	G2	2	3+
13	Positive, 80	Neg	12%	5%	T1, N0	IB	IDC	G2	3	2+
14	Neg, 0%, 3	2.1	20%	0%	T1, N0	I	IDC	G3	1	3+
15	Positive	Neg	4%	8%	T1, N1	IIA	Colloid	G2	4	3+
16	Positive	Neg	0%	5%	T1b,N1	IIA	IDC	G2	3	3+
17	Positive	Positive	4%	0%	T2, N1	IIB	ILC	G2	2	3+
18	Positive	Neg	18%	0%	T2, N1	IIB	IDC	G2	2	2+
19	Positive, 79	Neg	0%	0%	T2, N1	IIB	Tulular car	G1	4	2+
20	Positive	Neg	18%	9%	T2, N1	IIB	IDC	G2	2	3+
21	Positive	Neg	1%	3%	T2, N1	IIB	IDC	G2	2	2+
22	Positive	Neg	0%	0%	T1c, N1	IIA	IDC	G1	4	3+
23	Positive	Neg	13%	0%	T2, N1	IIB	IDC	G2	4	3+
24	Positive	Neg	10%	43%	T2, N1	IIB	IDC	G2	2	2+
25	Positive	Neg	8%	13%	T1c, N1	IIA	IDC	G2	4	2+
26	Positive	Neg	4%	0%	T2, N0	IIA	ILC	G2	2	2+
27	Positive	Neg	0%	0%	T2, N0	IIA	IDC	G2	4	3+
28	Positive, 0%	Positive	2%	4%	T2, N0	IIA	IDC	G3	1	1+
29	Positive 15	Positive	20%	2%	T2, N1	IIB	IDC	G2	3	3+
30	Positive, 0%	Neg	90%	0%	T2, N0	IIA	IDC	G3	3	2+
31	Positive	Neg	27%	25%	T2, N0	IIA	IDC	G2	1	2+
32	Positive	1.5	16%	0%	T2, N0	IIA	IDC	G2	4	3+

33	Neg, 0%, 1	0	5%	0%	T1, N1	IIA	ILC	G2	1	2+
34	Positive	Positive	9%	15%	T2, N3	IIIC	IDC	G2	2	3+
35	Positive, 35	Neg	3%	62%	T3, N2	IIIA	IDC	G2	2	3+
36	Positive	Positive	61%	32%	T3, N1	IIIB	IDC	G2	3	3+
37	Positive	Neg	9%	2%	T1, N2	IIIA	IDC	G2	4	3+
38	Positive	Neg	1%	73%	T2, N2	IIIA	IDC	G2	4	3+
39	Positive 12	Neg	11%	0%	T2, N3	IIIC	IDC	G2	2	3+
40	Positive	Neg	30%	25%	T4, Nx	IIIB?	ILC	G2	2	2+
41	Positive	Positive,	67%	92%	T3, N3	IIIC	IDC	G2	3	2+
42	Positive	Neg	54%	39%	T2, N2a	IIIA	Colloid	G1	3	2+
43	Positive	Neg	15%	39%	T4b, Nx	IIIB	IDC	G2	2	3+
44	Positive, 44	Positive	47%	0%	pT2, pN2	pIIIA	IDC	G3	1	2+
45	Positive, 97	Neg	59%	2%	T1c, N2a	IIIA	IDC	G2	1	2+
46	Positive, 94	Positive	58%	2%	T3, N2a	IIIA	IDC	G2	4	3+
47	Positive 80	0.5	18%	37%	pT2 N2	IIIA	IDC	G2	2	2+
